# Large-scale mitochondrial DNA analysis in Southeast Asia reveals evolutionary effects of cultural isolation in the multi-ethnic population of Myanmar

**DOI:** 10.1186/1471-2148-14-17

**Published:** 2014-01-28

**Authors:** Monika Summerer, Jürgen Horst, Gertraud Erhart, Hansi Weißensteiner, Sebastian Schönherr, Dominic Pacher, Lukas Forer, David Horst, Angelika Manhart, Basil Horst, Torpong Sanguansermsri, Anita Kloss-Brandstätter

**Affiliations:** 1 Division of Genetic Epidemiology, Innsbruck Medical University, Schöpfstraße 41, 6020 Innsbruck, Austria; 2 Institut für Humangenetik, Universität Münster, Münster, Germany; 3 Institute of Computer Science, University of Innsbruck, Innsbruck, Austria; 4 Institute of Pathology, LMU Munich, Munich, Germany; 5 Department of Dermatology, Columbia University, New York, NY, USA; 6 Department of Pediatrics, Chiang Mai University, Chiang Mai, Thailand

**Keywords:** Haplogroup, Complete mtDNA genome, Control region, Population genetics, Migration, Gene flow, Burma, Southeast Asia, Karen, Bamar, Demographic history

## Abstract

**Background:**

Myanmar is the largest country in mainland Southeast Asia with a population of 55 million people subdivided into more than 100 ethnic groups. Ruled by changing kingdoms and dynasties and lying on the trade route between India and China, Myanmar was influenced by numerous cultures. Since its independence from British occupation, tensions between the ruling Bamar and ethnic minorities increased.

**Results:**

Our aim was to search for genetic footprints of Myanmar’s geographic, historic and sociocultural characteristics and to contribute to the picture of human colonization by describing and dating of new mitochondrial DNA (mtDNA) haplogroups. Therefore, we sequenced the mtDNA control region of 327 unrelated donors and the complete mitochondrial genome of 44 selected individuals according to highest quality standards.

**Conclusion:**

Phylogenetic analyses of the entire mtDNA genomes uncovered eight new haplogroups and three unclassified basal M-lineages. The multi-ethnic population and the complex history of Myanmar were reflected in its mtDNA heterogeneity. Population genetic analyses of Burmese control region sequences combined with population data from neighboring countries revealed that the Myanmar haplogroup distribution showed a typical Southeast Asian pattern, but also Northeast Asian and Indian influences. The population structure of the extraordinarily diverse Bamar differed from that of the Karen people who displayed signs of genetic isolation. Migration analyses indicated a considerable genetic exchange with an overall positive migration balance from Myanmar to neighboring countries. Age estimates of the newly described haplogroups point to the existence of evolutionary windows where climatic and cultural changes gave rise to mitochondrial haplogroup diversification in Asia.

## Background

Myanmar (Burma), the largest country in Mainland Southeast Asia (SEA), covers an area of 676,578 km^2^ and is inhabited by ~55 million people. The fast evolutionary rate [1] and the non-recombining uniparental inheritance [2] of the mitochondrial DNA (mtDNA) generally qualifies mtDNA as highly potent marker for population and phylogenetic studies and mtDNA analyses have a long tradition in the exploration of human evolution [3]. Thanks to increasing knowledge on its mutation rate [4-7] mtDNA is also a valid tool for age estimates. Although Myanmar plays a crucial role for the population history of Southeast Asia [8], due to the long-lasting isolation of the country by its political regime, only very few mitochondrial DNA (mtDNA) data are available so far [9]. In order to close this gap on the genetic map of Southeast Asia, we collected DNA samples from 327 unrelated donors originating from 13 of the 14 political regions representing the most important ethnic groups of Myanmar and genotyped the entire mitochondrial control region (16024–16569; 1–576) of all samples and the entire mitochondrial genome of a subset of 44 selected samples.

This dataset from Myanmar is of great historic interest, because SEA is a key region of human population history with a first entry of anatomically modern humans of African descent about 60,000 years ago [10,11], who continued their way through the coastal route to Island SEA and Australia [8]. Following the glacial retreat in that area, also a north- and eastward migration towards the Yangtse and Yellow River basins of the ancestors of Sino-Tibetan tribes began [10]. So, also the initial colonization of China and the rest of East Asia had its origin in SEA [12,13]. Much later, probably driven by a Neolithic agricultural revolution, the Tibeto-Burman (Burmese-Lolo and Karen) branches of Sino-Tibetans moved back southwards through Yunnan to Myanmar and the SEA peninsula [11,14,15]. Ruled by changing kingdoms and dynasties [16], occupied by the British Empire (1824–1948) and lying on the trade route between India and China [17], Myanmar was influenced by a variety of cultures.

Analyzing mtDNA data from Myanmar is of great genetic interest, because in spite of accumulating knowledge in recent years [8,18-22] the resolution of the mitochondrial haplogroup phylogeny in SEA, especially in macrohaplogroup M, is still very low [23] compared to West-Eurasian haplogroups. Moreover, in population size analyses on mitochondrial DNA data, Atkinson et al. (2008) discovered that on the Indian subcontinent plus mainland SEA the first pronounced population expansion outside Africa took place around 52,000 years ago, and between 45,000 and 20,000 years before present the majority of the global population of Homo sapiens lived in that area [24].

Finally this dataset is also of sociocultural interest, because Myanmar is subdivided into more than 100 ethnic groups amongst them the Bamar represent 68% of the population. Other important minorities are Shan (10%), Karen (7%), Arakanese (4%), Chinese (3%) and the ethno-linguistically related Mon and Khmer (2% each). Since Myanmar’s independence from the British occupation, a lot of tensions emerged between the ruling Bamar and the remaining ethnic minorities, who suffered from government’s repression [25]. Amongst others especially the Karen people struggle against the domination of the Bamar culture [26].

With this study we expected to address the following historic questions: Is the changeful history of Myanmar reflected in its mitochondrial DNA diversity? How does its population structure fit into the overall picture of SEA? Was there a contribution of Indo-European language speakers to the genepool of Myanmar through the North Indian corridor or from the time as British colony? In addition, we aimed at contributing to the picture of human colonization of the Asian continent with the description and dating of new mitochondrial haplogroups. Finally, we were searching for mitochondrial genetic footprints of the ethnic heterogeneity of Myanmar and for signs of genetic isolation of individual ethnic groups.

## Results

### Haplogroup composition and new lineages

The study included 327 DNA samples from Myanmar citizens, who lived in Northern Thailand at the time of sample collection. The Myanmar sample, consisting of 327 mitochondrial control region sequences and 44 complete mitochondrial genomes, which constitute a subset of the 327 control region samples, exhibited pronounced mtDNA diversity displaying 113 distinct CR lineages, including eight in this study newly defined haplogroups and three different not classified basal M branches (Additional file 1: Table S1). F1a1a with 15.9% of all sequences was by far the most frequent haplogroup in this study, followed by C4b1 (7.0%), B6 (6.4%) and A4 (5.2%). R9b1a1a, D4 and G2b1a reached 4.6% each. The 78 individuals actually belonging to M split into 50 different haplogroups, 29 of them with only a single representative. The most common haplogroup in M was M21a (1.8%) (Additional file 1: Table S1).

**Figure 1:**
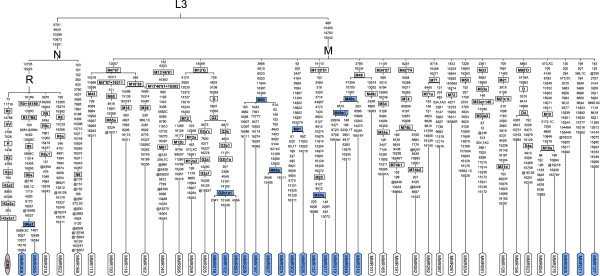
**Phylogenetic tree of 44 complete mitochondrial genomes.** The eight newly described haplogroups are shown in blue, and the corresponding samples as well as three new basal M-lineages (MMR127, MMR211 and MMR305) appear in blue, too. Mutations were annotated as differences to the rCRS; @…back mutation; LGM…last glacial maximum.

The 44 complete mitochondrial genomes revealed 11 so far undescribed mtDNA lineages, most of them lying within macrohaplogroup M (Figure 1). One lineage, comprising 3 haplotypes, clustered within haplogroup G2 and was termed G2b1a1 (MMR018, MMR083, MMR152). Two new lineages represented subgroups of haplogroup M49, and were named M49e (MMR310) and its subgroup M49e1 (MMR019, MMR049). Another lineage comprising 2 haplotypes was called M20a (MMR137, MMR317). Four haplotypes shared the mutations at positions 152 and 6253 with haplogroup M13′46′61 as defined in PhyloTree v.15 [27,28], but did not cluster consistently into this group in our phylogenetic analyses. Accordingly, we classified them as new haplogroup M90 (MMR187, MMR206), which was supported by 8 shared mutations and haplogroup M90a (MMR007, MMR225), which was supported by 13 additional mutations. Five more sequences branched at the very base of macrohaplogroup M, three of them as separate new lineages (MMR127, MMR211, MMR305) with only one representative, but one lineage composed of two haplotypes sharing 13 mutations with a previously published sequence (NCBI Accession Nr. HM030537) [8] and was therefore entitled as haplogroup M91 (MMR026, MMR302). One more mitochondrial lineage, a subgroup of B6a comprising of two haplotypes sharing 9 additional mutations was defined as new haplogroup B6a1 (MMR295, MMR308). The remaining 25 sequences could be assigned to existing haplogroups, but in most of them a lot of “private” mutations were observed (Figure 1) which probably will lead to new lineages with cumulating sequence information in future studies.

### Population structure of Myanmar

**Figure 2:**
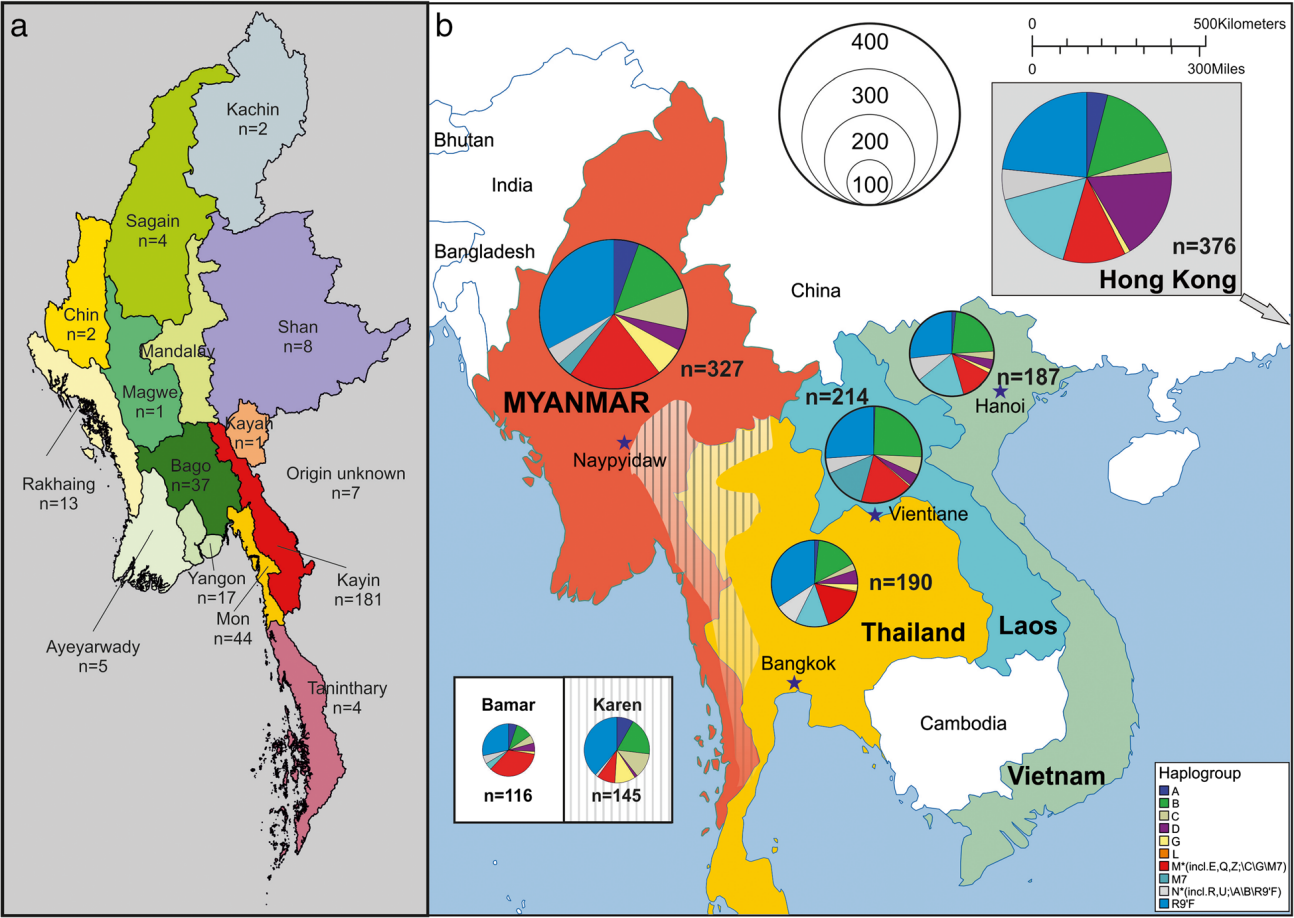
**Origin of samples and mitochondrial haplogroup distribution of Southeast Asian populations.** Although most of the study participants originated from Karen State (red), a broad sample spectrum from nearly all divisions and states of Myanmar **(a)** was included in this study. **b** shows the haplogroup distributions of populations from Myanmar and four other Southeast Asian regions. In the white insert box the haplogroup heterogeneity of two ethnic groups of Myanmar is illustrated. The hatched area in the map surrounding the border between Myanmar and Thailand shows the main population area of the Karen people. The Bamar represent the largest ethnic group (68%) in Myanmar. The size of the pie diagrams corresponds to sample size.

Out of the 14 provinces (7 states and 7 regions) of Myanmar, 13 provinces were represented in this study, the majority of samples coming from the Karen State. Detailed information on origin and cultural background of the sample donors is illustrated in Figure 2a and listed in Additional file 1: Table S1. Figure 2b illustrates the main haplogroup distribution of Myanmar compared to four other Southeast Asian regions: Thailand [29], Laos [23], Vietnam [30] and Hong Kong [31]. The Myanmar sample was typical for Southeast Asian populations with a high percentage of R9’F and B lineages as well as a variety of M haplogroups. The minor contribution of N lineages (without A, B and R9’F) to the gene pool also turned out to be characteristic for Southeast Asia. Noticeable was a relatively high percentage of A and C lineages in Myanmar compared to the neighboring countries (p < 0.001; Figure 2b). Within the Myanmar sample, two ethnic groups were predominant: the Bamar (35.47%) and the Karen (44.34%). Separate analyses of these two groups revealed that the observed over-representation of haplogroups A (A4, 8.28% of Karen) and C (C4b1, 12.41% of Karen) were mainly caused by the Karen population whereas the dominance of different M lineages (37.07% of Bamar, in no single HG more than 3 individuals) was much more pronounced in the Bamar population.

**Figure 3:**
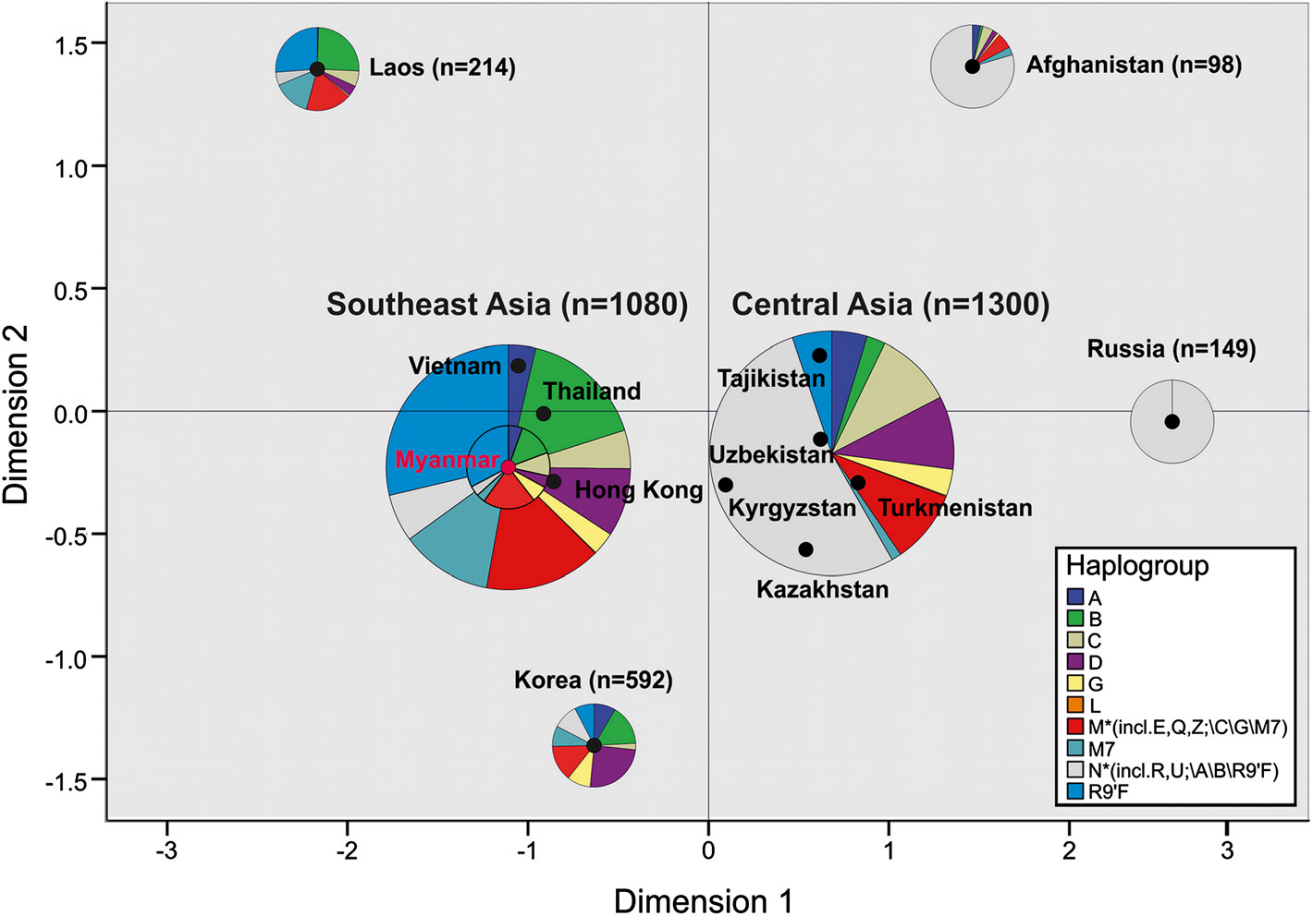
**Multi-dimensional scaling plot of pairwise Fst-values and haplogroup distribution of populations from Myanmar and 12 other Asian regions.** A distinct geographic pattern appeared in the multi-dimensional scaling plot (Stress = 0.086; R^2^ = 0.970) of pairwise Fst-values: The Myanmar sample fitted very well within the Southeast Asian cluster, the Central Asian populations formed a second cluster, the Korean sample represented East Asia, the Afghanistan population was representative for South Asia and Russia symbolized Western Eurasia. The main haplogroup distributions are displayed as pie charts. The size of the pie diagrams corresponds to sample size. The proportion of N-lineages (without A,B and R9’F) increases from very low percentages in Southeast and East Asia over 50% in Central Asia to more than 75% in Afghanistan and 100% in the sample of Russian origin. The proportion of the American founding haplogroups A,B,C and D displayed an interesting pattern: from inexistent in Russians it increased to more than 50% in East Asian Korea.

In the multi-dimensional scaling plot of pairwise F_ST_-values (listed in Additional file 2: Table S2) of populations from Myanmar and 12 other Asian regions a distinct geographical pattern appeared. The Myanmar sample fitted well within the Southeast Asian cluster, whereas the population sample from Laos appeared as an outlier (Figure 3). Central Asian populations formed a second cluster, the Korean sample represented East Asia, Afghanistan could be seen as South Asian and Russia as Western Eurasian representative. The haplogroup distribution of Myanmar was representative for the overall haplogroup distribution of Southeast Asia (pie charts, Figure 3). The distribution of N-lineages (without A, B and R9’F) was eye-catching with very low percentages in Southeast and East Asia, about 50% in Central Asia, more than 75% in Afghanistan and 100% in the sample of Russian origin. Also the proportion of the American founding haplogroups A, B, C and D displayed an interesting pattern: from inexistent in Russians, it appeared as a minor percentage in Afghanistan and became more abundant in Central Asia (25%) and Southeast Asia (35%) and had its climax with more than 50% in East Asian Korea.

### Comparison of the Bamar and the Karen people, two main ethnic groups in Myanmar

**Figure 4:**
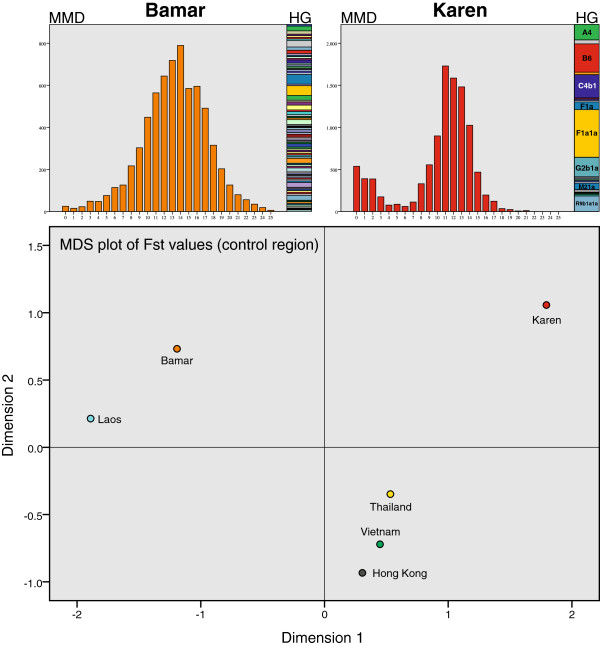
**Comparison of the Bamar and the Karen people, two main ethnic groups in Myanmar.** Pairwise mismatch distribution (MMD) plots (Figure 4, upper panel) indicated different demographic histories with signs of a strong and recent demographic expansion for the Bamar (orange), and evidence of a demographic equilibrium for the Karen people(red). The proportions of individual haplogroups of the two populations are depicted as bar charts next to the MMD-plots, with identical colors corresponding to identical haplogroups. In the lower panel, pairwise Fst-values of Bamar and Karen in comparison with four other Southeast Asian populations were visualized as multi-dimensional scaling (MDS) plot (Stress = 0.032; R^2^ = 0.995).

The sample within Myanmar turned out to be very inhomogeneous concerning the two different ethnic groups (Figure 4). Pairwise mismatch distribution plots indicated different demographic histories with signs of a strong and recent demographic expansion for the Bamar, and evidence of a demographic equilibrium for the Karen people. Within Karen we saw effects of genetic isolation from the mismatch distribution with many sequences showing minimal differences and also from the haplogroup composition with a high percentage of samples falling into identical haplogroups. In contrast, the haplogroup composition of Bamar was exceptionally diverse with 80 different haplogroups and a maximum of 6 samples in the same haplogroup (Figure 4). Multi-dimensional scaling plots of pairwise Fst-values (listed in Additional file 3: Table S3) from the two ethnic groups of Bamar and Karen and the four other Southeast Asian populations (Figure 4) showed that Thailand, Vietnam and Hong Kong appeared as a Southeast Asian cluster, whereas the two Burmese samples were far away from each other.

### Migration analyses

Migration analyses of Myanmar and four Southeast Asian regions displayed a vivid exchange of genetic material between the countries and demonstrated a strong outwards migration of Myanmar to all analyzed neighboring regions (for details see Additional file 4: Table S4). The migration rate from Myanmar to the neighboring country Thailand was 1.6-times higher than the migration rate from Thailand to Myanmar. Nearly twice as many people seemed to emigrate from Myanmar to Laos than Laotians immigrated to Myanmar. While the migration rates between Myanmar and Vietnam were nearly balanced, we observed a 1.3-time higher emigration rate from Myanmar to Hong Kong than from Hong Kong to Myanmar. Although the calculated migrations values and directions were not entirely consistent across the five analyses, in summary the effective migration rates characterized Myanmar genetically as an emigrating population.

### Age estimates of new haplogroups

The age estimates of the eight newly described haplogroups fell into four different periods. The oldest haplogroup M90 with an estimated age of 34,400 (95%-CI: 31,900 – 37,000) years dated in the Pleistocene, long before the last glacial maximum (LGM). The haplogroups M91 with an estimated age of 25,600 (95%-CI: 24,400 – 26,800) years and M49e (25,100; 95%-CI: 23,300 – 26,800 years) emerged at the beginning of the LGM, whereas the age of M49e1 (20,200; 95%-CI: 18,400 – 22,100 years) denoted the end of the LGM. The four remaining new haplogroups turned out to be much younger: B6a1 (10,200; 95%-CI: 9,800 – 10,600 years) and M20a (9,800; 95%-CI: 8,500 – 11,100 years) dated at the beginning of the Holocene, whereas the youngest new haplogroups M90a (4,800; 95%-CI: 4,300 – 5,200 years) and G2b1a1 (4,700; 95%-CI: 3,200 – 6,200 years) fell into the metal ages.

## Discussion

### Tracing the reasons for Myanmar’s extraordinarily diverse population structure

Myanmar turned out to be a hotspot for mitochondrial DNA diversity in Southeast Asia (SEA): The analyzed 327 mitochondrial control region sequences could be assigned to 113 different haplogroups (Additional file 1: Table S1) and from 44 complete mitochondrial genome sequences 11 new lineages emerged (Figure 1). This diversity can partly be explained by SEA’s long population history of over 60,000 years [8,12], and by the hypothesis that due to a Pleistocene population expansion, ~60% of the global human population lived in that area about 38,000 years ago [24]. Its geographic position on the trade route between the vast empires of India and China [17] and along the way of important migration events at several different time periods [8,13,18,32] could be another explanation for Myanmar’s haplogroup diversity. Furthermore, Burma’s population structure with more than 100 ethnicities scattered over a geographically differentiated country could have also contributed to its genetic variability. Such great variety of M-lineages as we found in Myanmar has only been described before in India [33]. Therefore, the prior hypothesis that the diversification of macrohaplogroup M originated in India [33] and that basal M-lineages spread to Myanmar could be extended by the theory that the radiation of M-lineages took place in a geographically wider area including Southeast Asia. Regarding the most frequent haplogroups, an interesting picture arose: In addition to the expected SEA groups [20,34] F1a1a (15.9%), R9b1a1a (4.6%), and M21a (1.8%) (Additional file 1: Table S1), the five other frequent mitochondrial haplogroups from our Burma sample (C4b1, B6, A4, D4 and G2b1a, together 27.8%) mainly represented Northeast and East Asian lineages [35-38]. B6 (6.4%) and D4 (4.6%), however, were also found in Island SEA [18,34,36]. A closer look on the newly described lineages (Figure 1) further supports an astonishing intermediate biogeographic position of Myanmar: M20a descends from a recently described haplogroup M20, found in Mainland SEA [32] and southern China [8]. Also the new lineage M91, directly descending from the M-node, has a previously detected member from southern China (NCBI Accession Nr. HM030537) [8]. The new haplogroup M49e and its subgroup M49e1, in contrast, derive from a lineage (M49) only described from tribal populations in Northeast India [33] and G2b1a1 as well as B6a1 arise from East Asian haplogroups [36,38]. The remaining new haplogroup M90 with its subgroup M90a as well as the three new lineages with only one representative support the assumption of a native emergence of basal M-lineages in this region. Despite the before mentioned specific features of Myanmar, the overall main haplogroup composition (Figure 2b) and the pairwise Fst-values (visualized as multidimensional scaling plot in Figure 3) characterize the country as a typical SEA population. The characteristic Mainland SEA haplogroups [20] B5a, F1a1a and M7b* were abundant in Myanmar (in sum 21.4%), only the M7-group was with 3.4% clearly under-represented compared to the four other SEA populations [23,29-31] included in this study (M7* between 12.7 and 18.2%). The further exceptions from SEA populations, namely the high percentages of the mainly East Asian haplogroups A and C (together 14.7% of the Myanmar sample) and the extraordinary diversity of superhaplogroup M*, could be attributed to the intrinsic characteristic of two ethnic groups: the over-representation of the haplogroups A4 (8.28%) and C4b1 (12.41%) in the Karen population compared to other Southeast Asian populations (p < 0.001) was the reason for the observed high percentage of East Asian haplogroups, and the large variety of haplogroup M in the Bamar population (37.07%) was responsible for the unexpected diversity of the M-cluster in Myanmar. Taken all together, Burma’s mitochondrial haplogroup composition, reflecting the country’s geographic position as well as its complex population structure, generally fits into the overall Southeast Asian picture, but adds important findings to it.

### Bamar and Karen share the same country but are genetically contrasting populations

The haplogroup composition of the two main ethnic groups in our study, the numerically (68% of the population) and politically dominating group in Myanmar, the Bamar, and the most repressed and also most separatist ethnicity, the Karen (7% of the population), differed enormously: The Bamar sample with its 80 different mitochondrial lineages was extraordinarily diverse, whereas almost three quarters (73.1%) of the Karen population could be assigned to only five haplogroups (Figure 4 upper panel).The multi-dimensional scaling plot of Fst-values (Figure 4 lower panel) underlined those differences. Pairwise mismatch distribution plots showed signs of a recent demographic expansion for the Bamar, but demographic equilibrium and many sequences with minimal differences in the Karen population pointed to effects of genetic isolation (Figure 4 upper panel). The conclusion that Bamar and Karen differ genetically is not new: a study on CYP2C19*3 allele frequencies, an important gene for the response to barbiturates and malaria drugs [39] and a survey on heme-oxygenase 1 promoter polymorphisms [40] found significant differences between participants of Bamar or Karen origin. These findings could be interpreted both historically and by recent sociocultural aspects of present-time Myanmar: Although Bamar and Karen both are of Tibeto-Burman origin and derive from ancient tribes of northwestern China [14], their history differs: the Bamar migrated from Yunnan/China into the Irrawaddy valley in the 7th century AD, thereby replacing and absorbing the original tribes of Pyu and Mon [41]. In the course of that absorption they presumably “incorporated” older local haplogroups [14]. The ethnic origin of the Karen and the migration routes before their arrival in Myanmar around the sixth century AD is largely unknown [42]. Their own legends tell about an ancestry from a “sandy region in the North”, sometimes interpreted as the Gobi Desert, but a more plausible explanation includes an origin in the Yellow River region, and a migration over Yunnan along the Salween or Mekong into today’s Shan state and then into the mountain regions farther south (now Kayin State) [42,43]. The Karen claim to be amongst the first settlers of Myanmar and despite their diverse linguistic and cultural subgroups, their different religions and different geographic locations, they define themselves as unity with a clear dissociation from the Bamar [43]. Suppression and displacement of Karen and other peoples were already documented in the 18th century [44], and many Karen fled and still flee to Thailand [43]. Even until very recently, there was an armed conflict between the Bamar government and the Karen National Union in Myanmar [26]. Another reason for the observed dissimilarity between those two ethnic groups could be the traditionally matrilocal and matrilineal culture of the Karen [26,42], which cannot be found in the Bamar. While the haplogroup diversity for mitochondrial DNA is supposed to be generally lower within matrilocal groups than within patrilocal groups and vice versa for the Y-chromosome [45], an analogous pattern in genetic diversity was not confirmed with Y-chromosome data, where Burmese-Lolo (i.e. Bamar) and Karen were shown to exhibit similar haplotype patterns [11]. In summary, the distinct genetic dissimilarity between Bamar and Karen seems to result, besides their different population sizes, not so much from geographic separation of the two ethnic groups, but from divergent population histories and even more from their different cultural traditions and social constraints.

### Can the genetic influence of Myanmar on its neighboring countries be explained by their shared ancestry alone or is it partially caused by politically forced migration events?

The recent history of Myanmar, with its isolation politics lasting for decades, resulted in a very low published immigration estimate of 0.23 persons per 1000 inhabitants and a net emigration estimate of 0.3/1000 migrants per year (CIA World fact book 2013, https://www.cia.gov/library/publications/the-world-factbook). Therefore, the genetically manifested vivid exchange of Myanmar with neighboring regions and an emigration exceeding immigration by over 30% (Additional file 1: Table S4), is an interesting finding. The magnitude of the observed migration rates could reflect the widely shared population history of Myanmar, Thailand, Laos, Vietnam and South China, all descending from Sino-Tibetan tribes [11,14,15]. However, the majority of the present genetic exchange, particularly with the Thai population, seems to originate from the Karen minority: The Thai samples included in this study come from the Chiang Mai region [29], where traditionally many Karen live, nowadays a considerable proportion of them as refugees [46]. Migration analyses, especially the dominating outwards migration, could therefore also indicate an active marginalization and displacement of ethnic minorities, especially of the Karen people with ~140,000 Karen refugees alone in Thailand.

### Did climate change and cultural inventions influence human population history in Mainland Southeast Asia?

The timing of the most recent common ancestor of the youngest newly described haplogroups M90a (mean time estimate 4,800 years) and G2b1a1 (4,700 years) falls into a time of cultural change. An agricultural revolution in southern China with subsequent expansion to Mainland and Island SEA took place around that time [34]. Haplogroup G2b1a1, originating from East Asian lineages [36,38] would perfectly trace this migration wave. The emerging of haplogroups M20a and B6a1 in the early Holocene, together with F1a1a also appearing in Indochina at the same time [18], fits perfectly into a time of a supposed expansion of people from Mainland SEA and southern China, possibly driven by global warming and sea level rises, to Island SEA [18,32,34,47,48]. The potential Northeast Indian haplogroup M49e (25,100 years) and its subgroup M49e1 (20,200 years) appear at the beginning, respectively versus the end of the LGM, which would be a typical coalescent time of East Asian lineages in Northeast Asia [33]. Also the new Southeast Asian lineage M91 (25,600) dates at the beginning of the LGM together with R9b, another lineage from Indochina [34]. It is not clear what could have driven a diversification of mitochondrial haplogroups in SEA during the LGM; perhaps an extension of settlement area to the current islands of Sumatra, Java and Borneo, being parts of the Asian mainland at that time, could serve as an explanation. About the emerging of haplogroup M90 (34,400 years) in the Pleistocene and the additional three undated new basal M-lineages we can only speculate that the proposed population expansion in South and Southeast Asia between 45,000 and 20,000 years before present [24] resulted in new haplogroups. The age estimates of our eight newly described haplogroups, together with previously published coalescence times of other lineages, point to the existence of distinct “evolutionary time slots” where climatic as well as cultural changes gave rise to mitochondrial haplogroup diversification in Southeast Asia.

### Strengths and limitations

This survey provides detailed insight into human mitochondrial DNA evolution in Myanmar for the first time, and thereby sheds more light onto a crucial stage of Southeast Asian population history. Our study collection was selected with the aim of covering a maximum of Myanmar’s diversity; indeed, it included samples from every political region except Mandalay division, and from 11 different ethnicities including the numerically most important. At the same time it provides sufficient sample depth to address ethnicity specific questions in the case of Bamar and Karen, and therefore optimizes the information content within the limits of a manageable sample size. Another important strength of this study represent the personal interviews with each study participant following a sophisticated questionnaire and therefore making assignment problems or misinterpretations by third persons very unlikely. Thanks to this questionnaire (Additional file 5: Table S5), we were able to obtain reliable information on age, sex, specific ethnicity and geographic origin of the participants, and in addition also the places of birth of maternal ancestors of the preceding two generations were compiled.

However, a major limitation of our study is that samples were collected from Myanmar citizens living in Northern Thailand at the time of sample collection, and were not collected in Myanmar *per se*. In addition, our dataset still suffers, compared to the actual demographic situation of Myanmar, from an uneven sample distribution with an under-representation of the populous Irrawaddy basin. For important ethnic groups like Shan, Arakanese, Mon and Chin, sample sizes were too small to address sub-population specific questions. A second caveat of this study in regard of demographic estimates is that mtDNA, even the complete mtDNA genome, only represents a single locus. We negotiated those shortcomings by specifically addressing the Bamar as the dominant group in Myanmar, and the Karen, representing the most persecuted and most separatist minority in Myanmar, for population genetic analyses. We restricted the interpretation of the demographic analysis results to migration rates, where an estimate based on one locus is valid [49].

### Conclusions and outlook

The multi-ethnic population and the complex history of Myanmar were well reflected in its distinct mitochondrial DNA heterogeneity. The mitochondrial haplogroup distribution in Myanmar showed a typical Southeast Asian pattern, confirming earlier findings but also adding new information: the population sample of Myanmar displayed quite a few parallels to North and Northeast Asian and also to South Asian populations. No traces of European or African influence to the maternal gene pool of Myanmar were detected. The population structure of the extraordinarily diverse Bamar differed substantially from that of the Karen people who displayed signs of genetic isolation. These results, together with the genetically manifested net outwards migration to neighboring regions, raise questions about the sociocultural circumstances in Myanmar. The description and dating of eight new mitochondrial haplogroups and the detection of three further basal M lineages shed more light on the population history of Southeast Asia.

This study resulted in important findings, but, as always with gaining knowledge, further questions arise: Does the genetic pattern of other ethnic groups in Myanmar, respectively in Southeast Asia, display such great differences as Bamar and Karen? What does this imply in terms of cultural and social issues? Is the exceptional haplogroup diversity found in Bamar so very special in this region and if this is the case, what lies behind that? How would the overall picture of a detailed study from a country in the same region, but with a more homogeneous ethnic structure differ from that of Myanmar? To address these and many more questions, it seems worth spending time on developing sound study designs with meaningful questionnaires going into detail in terms of biogeographic and cultural population characteristics like the presence and distribution of ethnic groups. Furthermore, the phylogeny of the human mitochondrial genome, especially in macrohaplogroup M, is far from being exhaustively investigated and a plethora of new lineages still await to be discovered in future studies. The use of next generation sequencing technologies will help to obtain more complete mitochondrial genome data in a shorter time span, but one always has to keep an eye on highest sequence quality, a prerequisite for indisputable findings.

## Conclusions

Phylogenetic analyses of 44 entire mtDNA genomes uncovered eight new haplogroups and three unclassified basal M-lineages. The multi-ethnic population and the complex history of Myanmar were reflected in its mtDNA heterogeneity. Population genetic analyses of Burmese control region sequences combined with population data from neighboring countries revealed that the Myanmar haplogroup distribution showed a typical Southeast Asian pattern, but also Northeast Asian and Indian influences. The population structure of the extraordinarily diverse Bamar differed from that of the Karen people, who displayed signs of genetic isolation. Migration analyses indicated a considerable genetic exchange with an overall positive migration balance from Myanmar to neighboring countries. Age estimates of the newly described haplogroups point to the existence of evolutionary windows where climatic and cultural changes gave rise to mitochondrial haplogroup diversification in Asia.

## Methods

### DNA samples and sequencing

Blood samples were collected from 327 unrelated Burmese individuals, who came from 13 of the 14 regions of Myanmar, but lived in Thailand at the time of the sample collection. The study was approved by the Thai Ministry of Public Health in accordance with international ethical standards. According to the Declaration of Helsinki, participation in the study was on voluntary basis and informed consent was obtained from all donors (Chiang Mai University, Thailand, January 9, 2008). Detailed information on the geographical origin and ethnic background of the samples including the maternal region of was collected from all samples can be found in Figure 2 and Additional file 1: Table S1. An example of such a questionnaire on maternal origin is given in Additional file 5: Table S5. Genomic DNA was extracted on a BioRobot EZ1 advanced Workstation (QIAGEN, Hilden, Germany) and quantified on an Infinite® 200 NanoQuant (Tecan Group Ltd., Männedorf, Switzerland). Mitochondrial DNA control region (CR; nucleotide positions 16024–16569; 1–576) sequences were obtained following the protocol described in Brandstätter et al. [50]. To refine haplogroup affiliations we additionally sequenced informative coding region segments in 24 samples (listed in Additional file 1: Table S1). In addition, we performed complete mitochondrial genome sequencing as described in Kloss-Brandstätter et al. [51] in order to refine the phylogeny of 44 samples that could not be assigned to a haplogroup more specific than paragroups M* or N*.

### Sequence evaluation, quality assurance and haplogroup assignment

Sequence electropherograms were aligned to the revised Cambridge Reference Sequence (rCRS; NC_012920) [52] and evaluated independently by two different mtDNA technicians with the sequence analysis software Sequencher (v5.0, GeneCodes, Ann Arbor, MI). Despite a recent suggestion to replace the rCRS by a Reconstructed Sapiens Reference Sequence (RSRS; [53]), we prefer to maintain the rCRS as proposed by many mtDNA scientists [54,55]. Validation was performed by a senior mtDNA scientist using the mtDNA management software eCOMPAGT [56]. MtDNA haplotypes were assigned to haplogroups based on PhyloTree v.15 [27,28] with HaploGrep [57].

### Population genetic analysis

In order to shed light on the genetic structure of Burma and its phylogeographic position within Southeast Asia, three different datasets were compiled: the first dataset comprised 1294 Southeast Asian samples consisting of samples from this study (n = 327), Hong Kong (n = 376) [31], Thailand (n = 190) [29], Vietnam (n = 187) [30] and Laos (n = 214) [23]. In the second dataset, the geographic range was expanded with additional data from Central Asia (n = 1550; including samples from Uzbekistan, Kazakhstan, Kyrgyzstan, Russia, Afghanistan, Turkmenistan, and Tajikistan) [58] and East Asia (n = 592) [59]. In the third dataset, we split the Myanmar sample and analyzed the ethnic groups of Bamar (n = 116) and Karen (n = 145) in comparison with other Southeast Asian populations. ARLEQUIN version 3.5.1.3 [60] was used for the calculation of mismatch distributions, molecular diversity indices, and analyses of molecular variance (AMOVA) with entire control region sequences (16024–576) excluding C-insertions on positions 16193, 309, 315 and 573.

### Statistical methods

For visualizing the AMOVA results, multi-dimensional scaling plots based on pairwise Fst-values were generated with PASW Statistics 18 (SPSS Inc.). Differences in haplogroup-distributions between different populations were evaluated with a Chi-Square Test.

### Phylogenetic analyses and age estimates

For phylogenetic tree reconstruction of the 44 complete mitochondrial genomes we included the rCRS [52]. The best fitting model of evolution selected by Modeltest as implemented in the computer program MEGA 5 [61] was Tamura-Nei 1993 [62] with Gamma distribution and invariant sites (TN + G + I). The evolutionary history was inferred with the Maximum Likelihood method using the TN + G + I model in MEGA 5. Initial trees for the heuristic search were obtained by applying the Neighbor-Joining method [63] to a matrix of pairwise distances estimated using the Maximum Composite Likelihood (MCL) approach. All positions containing gaps and missing data were excluded. Phylogenetic trees were also generated using Markov-Chain-Monte-Carlo (MCMC) sampling with the computer program BEASTMC3 v1.7.4 [64]. Analyses were performed with two different models of evolution, TN + G [62] and HKY [65], and the “tree prior” was set to coalescent, Bayesian skyline with 10 groups and a constant skyline model. One cold and two hot chains were run with the temperatures of 0.5 and 0.333 and a chain swapping at every 100 generations. 20,000,000 generations were sampled every 2000 steps and the first 10% of generations were regarded as burn-in. Each log file was reviewed in TRACER 1.5 [66] for the stability of MCMC chains [67] and the tree files were combined with TreeAnnotator v.1.7.5 included in the BEAST package [64]. For all MEGA and BEAST analyses, a constant clock with a mutation rate of 1.665 × 10^-8^ base substitution per nucleotide per year [4] for the complete mitochondrial genome was applied. Mean age estimates and for each new haplogroup were calculated as arithmetic means from the singular age estimates obtained from the five different phylogenetic analyses (two repetitions of MEGA, three repetitions of BEAST). The 95%-confidence intervals were obtained from the arithmetic means and corresponding standard deviations.

### Estimation of migration rates

Migration rates, defined as M = m/μ, where M is the effective migration rate, m the immigration rate per generation, and μ is the mutation rate per generation and site, between Myanmar and four other Southeast Asian countries (Hong Kong, Thailand, Vietnam and Laos) were inferred with MIGRATE version 3.4.2 [68,69] using coalescence theory. In order to get an unbiased estimate of the mutation rate, a subsample of 180 randomly chosen sequences per population was used. MIGRATE uses both Markov Chain Monte Carlo-based (MCMC) Bayesian and Maximum Likelihood (ML) procedures to calculate population genetic parameters [70]. In this study we applied the ML approach assuming a constant mutation rate; 10 short chains and 1 long chain were run, each with a sampling increment of 100; the short chains sampled 50,000 genealogies and the long chain sampled 500,000 genealogies, all with a “burn-in” of 10,000. “Heating” (Metropolis-Coupled MCMC; “MCMCMC”) with four chains (temperatures 1.0, 1.5, 3, and 1,000,000) and a static heating scheme was applied. Migrate analyses were repeated four times with independent random sampling of sequences. In addition, Lamarc-2.1.8 (Maximum Likelihood Parameter Estimation using Hastings-Metropolis Markov Chain Monte Carlo) [49] was used to estimate migration rates, using the information of 50 randomly picked mitochondrial control region sequences per population. Maximum likelihood analyses (F84 base substitution model, base frequencies estimated, TI/TV ratio = 2) were performed with 10 initial and 2 final Markov chains sampling 500 respectively 10,000 trees, with a sampling increment of 20, a burn-in of 1000 and chain temperatures of 1, 1.1 and 1.2. For the final estimate of the migration rates between the selected populations, the results of each and every analysis were averaged.

### Data access

All sequences from Myanmar were submitted to NCBI GenBank (http://www.ncbi.nlm.nih.gov/genbank); 327 mitochondrial control region sequences are available under the Accession Numbers JX288765-JX289091 and 44 complete mitochondrial genomes are available under the Accession Numbers JX289092-JX289135. The assignment of all samples to their sequence Accession Numbers is listed in Additional file 1: Table S1.

## Competing interests

The authors declare that they have no competing interests.

## Authors’ contributions

MS participated in sequence alignment, performed phylogenetic analyses, created figures and wrote the paper. JH, DH, BH and TS collected the samples, performed interviews on the origin of samples, and extracted the DNA. GE and AM did the lab work including PCR, Sanger sequencing and sequence alignment. HW, SS, DP and LF performed phylogenetic analyses and created figures. AK-B initiated the study, validated the independent sequence alignments, controlled the phylogenetic analyses, created figures and wrote the paper. All authors read and approved the final manuscript.

## Supplementary Material

Additional file 1: Table S1 Detailed description of the analyzed samples, including ethnicities, places of birth, Genbank accession numbers, mtDNA profiles, and mtDNA haplogroup affiliations. Click here for file

Additional file 2: Table S2 Results of AMOVA (Analysis of Molecular Variance) of selected Asian populations. Click here for file

Additional file 3: Table S3 Results of AMOVA (Analysis of Molecular Variance) of Myanmar and SEA. Click here for file

Additional file 4: Table S4 Estimation of effective migration rates (M = m/μ) between. Myanmar and four other Southeast Asian regions. Click here for file

Additional file 5: Table S5 Example of a questionnaire used during sample collection. Click here for file
