# Supplementary material for: Large-scale mitochondrial DNA analysis in Southeast Asia reveals evolutionary effects of cultural isolation in the multi-ethnic population of Myanmar
Source: BMC Evol Biol. 2014 Jan 28;14:17. doi: 10.1186/1471-2148-14-17 (PMC3913319; doi:10.1186/1471-2148-14-17)
Supplement: Additional file 2: Table S2 — Results of AMOVA (Analysis of Molecular Variance) of selected Asian populations. [file 1471-2148-14-17-S2.pdf]

Supplemental Table S2: Results of AMOVA (Analysis of Molecular Variance) of selected Asian populations

a) Design and results:

Source of variation	d.f.	Sum of squares	Variance components	Percentage of variation
Among populations	12	859.455	0.25314 Va	4.15
Within populations	3,423	20,003.602	5.84388 Vb	95.85
Total	3,435	20,863.057	6.09702	100

b) Population pairwise F_{STs}

Distance method: Pairwise difference

	Afghanistan	Myanmar	Hong Kong	Uzbekistan	Kazakhstan	Kyrgyzstan	Laos	Korea	Russians from Uzbekistan	Tajikistan	Thailand	Turkmenistan	Vietnam
Afghanistan	0												
Myanmar	0.08576	0											
Hong Kong	0.07269	0.02439	0										
Uzbekistan	0.0356	0.04036	0.03206	0									
Kazakhstan	0.05053	0.04104	0.02778	0.00452	0								
Kyrgyzstan	0.05377	0.03228	0.01627	0.00858	0.00525	0							
Laos	0.11761	0.06393	0.05454	0.086	0.08941	0.06886	0						
Korea	0.09431	0.04769	0.01435	0.03642	0.02334	0.01249	0.08284	0					
Russians from Uzbekistan	0.05619	0.10209	0.09732	0.03454	0.05532	0.06343	0.13645	0.11739	0				
Tajikistan	0.04877	0.04231	0.03759	0.01619	0.02435	0.02189	0.08081	0.05437	0.05434	0			
Thailand	0.07087	0.01296	0.00572	0.03317	0.03354	0.02376	0.05058	0.03328	0.08905	0.03459	0		
Turkmenistan	0.04429	0.04964	0.03965	0.00333	0.00775	0.01183	0.09269	0.04232	0.03333	0.01982	0.04189	0	
Vietnam	0.0734	0.01904	0.00671	0.04005	0.04399	0.02799	0.04485	0.03517	0.09813	0.03961	0.00542	0.04937	0

c) Fst p-values (significant p-value after Bonferroni correction 0.000641)

	Afghanistan	Myanmar	Hong Kong	Uzbekistan	Kazakhstan	Kyrgyzstan	Laos	Korea	Russians from Uzbekistan	Tajikistan	Thailand	Turkmenistan	Vietnam
Afghanistan	*												
Myanmar	<0.0001	*											
Hong Kong	<0.0001	<0.0001	*										
Uzbekistan	<0.0001	<0.0001	<0.0001	*									
Kazakhstan	<0.0001	<0.0001	<0.0001	<0.0001	*								
Kyrgyzstan	<0.0001	<0.0001	<0.0001	<0.0001	0.00098	*							
Laos	<0.0001	<0.0001	<0.0001	<0.0001	<0.0001	<0.0001	*						
Korea	<0.0001	<0.0001	<0.0001	<0.0001	<0.0001	<0.0001	<0.0001	*					
Russians from Uzbekistan	<0.0001	<0.0001	<0.0001	<0.0001	<0.0001	<0.0001	<0.0001	<0.0001	*				
Tajikistan	<0.0001	<0.0001	<0.0001	<0.0001	<0.0001	<0.0001	<0.0001	<0.0001	<0.0001	*			
Thailand	<0.0001	<0.0001	0.00098	<0.0001	<0.0001	<0.0001	<0.0001	<0.0001	<0.0001	<0.0001	*		
Turkmenistan	<0.0001	<0.0001	<0.0001	0.00098	<0.0001	<0.0001	<0.0001	<0.0001	<0.0001	<0.0001	<0.0001	*	
Vietnam	<0.0001	<0.0001	0.00098	<0.0001	<0.0001	<0.0001	<0.0001	<0.0001	<0.0001	<0.0001	0.01660	<0.0001	*

d) Population average pairwise differences (16024-16569;1-576; C-insertions around 16193,309,315 and 573 were ignored)

Above diagonal: Average number of pairwise differences between populations (PiXY)

Diagonal elements: Average number of pairwise differences within populations (PiX)

Below diagonal: Corrected average pariwise difference (PiXY-(PiX+PiY)/2)

Distance method: Pairwise difference

	Afghanistan	Myanmar	Hong Kong	Uzbekistan	Kazakhstan	Kyrgyzstan	Laos	Korea	Russians from Uzbekistan	Tajikistan	Thailand	Turkmenistan	Vietnam
Afghanistan	10.40143	11.87796	12.20527	11.27253	11.58257	13.00753	13.36978	11.99111	10.1215	12.0471	12.17234	10.99285	12.07781
Myanmar	10.4168	11.27113	12.00311	11.76914	11.90342	13.10447	12.98383	11.8268	11.1953	12.38692	11.8717	11.51238	11.82761
Hong Kong	0.93078	0.29377	12.14755	12.12204	12.19479	13.33988	13.32419	11.8696	11.65008	12.78469	12.22844	11.85414	12.12494
Uzbekistan	0.41329	0.47505	0.38975	11.31704	11.49051	12.81936	13.31868	11.71199	10.40367	12.08397	12.14136	10.99841	12.10871
Kazakhstan	0.60165	0.48765	0.3408	0.05178	11.56041	12.90164	13.51424	11.67823	10.76381	12.31156	12.2749	11.16953	12.28862
Kyrgyzstan	0.75288	0.41497	0.21217	0.1069	0.0675	14.10787	14.59292	12.83063	12.24216	13.58368	13.46264	12.50457	13.40377
Laos	1.63756	0.81676	0.71891	1.12866	1.20253	1.00748	13.06301	13.21593	12.68184	13.88403	13.29016	13.03417	13.08996
Korea	1.16309	0.56393	0.16852	0.42616	0.27072	0.14939	1.05712	11.25461	11.42748	12.52473	12.10551	11.41973	12.01161
Russians from Uzbekistan	0.5558	1.19475	1.21131	0.38016	0.61861	0.82323	1.78535	1.43518	8.72998	11.23428	11.49299	10.01107	11.47951
Tajikistan	0.61441	0.51938	0.47893	0.19348	0.29938	0.29776	1.12054	0.66545	0.63731	12.46396	12.75876	11.7675	12.70613
Thailand	0.88683	0.15134	0.06987	0.39805	0.4099	0.32391	0.67386	0.39341	1.04321	0.44199	12.16959	11.8802	12.11987
Turkmenistan	0.48945	0.57413	0.47768	0.0372	0.08664	0.14794	1.19997	0.48974	0.3434	0.23283	0.49271	10.60538	11.85202
Vietnam	0.90777	0.22273	0.08184	0.48087	0.53909	0.38051	0.58913	0.41498	1.1452	0.50483	0.06575	0.58001	11.93865

Sources of data taken from literature:

Afghanistan	Irwin et al.: **The mtDNA composition of Uzbekistan: a microcosm of Central Asian patterns.** Int J Legal Med 2010, 124: 195-204.
Hong Kong	Irwin et al.: **Mitochondrial DNA control region variation in a population sample from Hong Kong, China.** Forensic Sci Int Genet 2009, 3: e119-e125.
Uzbekistan	Irwin et al.: **The mtDNA composition of Uzbekistan: a microcosm of Central Asian patterns.** Int J Legal Med 2010, 124: 195-204.
Kazakhstan	Irwin et al.: **The mtDNA composition of Uzbekistan: a microcosm of Central Asian patterns.** Int J Legal Med 2010, 124: 195-204.
Kyrgyzstan	Irwin et al.: **The mtDNA composition of Uzbekistan: a microcosm of Central Asian patterns.** Int J Legal Med 2010, 124: 195-204.
Laos	Bodner et al.: **Southeast Asian diversity: first insights into the complex mtDNA structure of Laos.** BMC Evol Biol 2011, 11:49.
Korea	Lee et al.: **Mitochondrial DNA control region sequences in Koreans: identification of useful variable sites and phylogenetic analysis for mtDNA data quality control.** Int J Legal Med 2006, **120:** 5-14.
Russians from Uzbekistan	Irwin et al.: **The mtDNA composition of Uzbekistan: a microcosm of Central Asian patterns.** Int J Legal Med 2010, 124: 195-204.
Tajikistan	Irwin et al.: **The mtDNA composition of Uzbekistan: a microcosm of Central Asian patterns.** Int J Legal Med 2010, 124: 195-204.
Thailand	Zimmermann et al.: **Forensic and phylogeographic characterization of mtDNA lineages from northern Thailand (Chiang Mai).** Int J Legal Med 2009, 123: 495-501.
Turkmenistan	Irwin et al.: **The mtDNA composition of Uzbekistan: a microcosm of Central Asian patterns.** Int J Legal Med 2010, 124: 195-204.
Vietnam	Irwin et al.: **Mitochondrial control region sequences from a Vietnamese population sample.** Int J Legal Med 2008, 122: 257-259.
