# Supplementary material for: Large-scale mitochondrial DNA analysis in Southeast Asia reveals evolutionary effects of cultural isolation in the multi-ethnic population of Myanmar
Source: BMC Evol Biol. 2014 Jan 28;14:17. doi: 10.1186/1471-2148-14-17 (PMC3913319; doi:10.1186/1471-2148-14-17)
Supplement: Additional file 3: Table S3 — Results of AMOVA (Analysis of Molecular Variance) of Myanmar and SEA. [file 1471-2148-14-17-S3.pdf]

Supplemental Table S3: Results of AMOVA (Analysis of Molecular Variance) of Myanmar and SEA

a) Design and results:

Source of variation	d.f.	Sum of squares	Variance components	Percentage of variation
Among populations	5	248,302	0.22044 Va	3.42
Within populations	1,222	7,387.606	6.04550 Vb	96.48
Total	1,227	7,635.908	6.26595	100

b) Population pairwise Fsts

Distance method: Pairwise difference

	Karen	Bamar	Hong Kong	Thailand	Vietnam	Laos
Karen	0					
Bamar	0.06007	0				
Hong Kong	0.05115	0.04163	0			
Thailand	0.03016	0.03624	0.00587	0		
Vietnam	0.04013	0.04314	0.00666	0.00527	0	
Laos	0.07959	0.01629	0.05305	0.04549	0.04413	0

c) Fst p-values (significant p-value after Bonferroni correction: 0.0033)

	Karen	Bamar	Hong Kong	Thailand	Vietnam	Laos
Karen	*					
Bamar	<0.0001	*				
Hong Kong	<0.0001	<0.0001	*			
Thailand	<0.0001	<0.0001	0.00195	*		
Vietnam	<0.0001	<0.0001	0.00195	0.01172	*	
Laos	<0.0001	<0.0001	<0.0001	<0.0001	<0.0001	*

d) Population average pairwise differences (16024-16569;1-576; C-insertions around 16193,309,315 and 573 were ignored)

Above diagonal: Average number of pairwise differences between populations (PiXY)

Diagonal elements: Average number of pairwise differences within populations (PiX)

Below diagonal: Corrected average pariwise difference (PiXY-(PiX+PiY)/2)

	Karen	Bamar	Hong Kong	Thailand	Vietnam	Laos
Karen	10.2567	12.51024	11.74897	11.5349	11.5124	12.69513
Bamar	0.73923	13.28531	13.16903	13.16593	13.11543	13.39127
Hong Kong	0.62442	0.53018	11.9924	12.12034	11.99312	13.22012
Thailand	0.35331	0.47003	0.0709	12.10649	12.03338	13.18618
Vietnam	0.46737	0.5561	0.08024	0.06345	11.83336	13.02529
Laos	1.03527	0.21712	0.69242	0.60143	0.5771	13.06301
