# Supplementary material for: Large-scale mitochondrial DNA analysis in Southeast Asia reveals evolutionary effects of cultural isolation in the multi-ethnic population of Myanmar
Source: BMC Evol Biol. 2014 Jan 28;14:17. doi: 10.1186/1471-2148-14-17 (PMC3913319; doi:10.1186/1471-2148-14-17)
Supplement: Additional file 4: Table S4 — Estimation of effective migration rates (M = m/μ) between. Myanmar and four other Southeast Asian regions. [file 1471-2148-14-17-S4.pdf]

Supplemental Table S4: Estimation of effective migration rates (M=m/μ) between Myanmar and four other Southeast Asian regions

a) outgoing M from Myanmar to

	Migrate_1	Migrate_2	Migrate_3	Migrate_4	Lamarc_1	mean	STD
Hong Kong	307.57	188.41	464.3	484.62	123.94	313.77	160.97
Thailand	431.33	281.03	524.72	614.75	149.91	400.35	186.65
Vietnam	527.54	251.12	174.99	120.91	77.3	230.37	178.34
Laos	246.97	379.03	409.57	480.92	268.6	357.02	98.14

b) incoming M to Myanmar from

	Migrate_1	Migrate_2	Migrate_3	Migrate_4	Lamarc_1	mean	STD
Hong Kong	105.19	434.17	334.69	209.75	85.64	233.89	149.5
Thailand	275.48	364.2	311.76	197.81	123.36	254.52	95.08
Vietnam	161.21	172.57	202.32	179.63	264.06	195.96	40.93
Laos	102.73	319.8	326.36	82.29	64.26	179.09	132.17
