# Supplementary material for: Large-scale mitochondrial DNA analysis in Southeast Asia reveals evolutionary effects of cultural isolation in the multi-ethnic population of Myanmar
Source: BMC Evol Biol. 2014 Jan 28;14:17. doi: 10.1186/1471-2148-14-17 (PMC3913319; doi:10.1186/1471-2148-14-17)
Supplement: Additional file 5: Table S5 — Example of a questionnaire used during sample collection. [file 1471-2148-14-17-S5.pdf]

Supplemental Table S5: Example of a questionnaire used during sample collection

(2)

NO.	REL (Relation / ญาติ)	GEN (Gender)	AGE	~~NAME~~ ETHNICITY	POB (Place of Birth)	PR #.	MOMs POB	M PR #.	MOMs MOMs POB	M M PR #.
11		M	24.	W	Loi Kaw	10	Loi Kaw	10	Loi Kaw	10
12		M	24	J	ต่อแลลี้	13	ต่อแลลี้	13	ต่อแลลี้	13
13		F	19	J	ต่อแลลี้	13.	ต่อแลลี้	13	ต่อแลลี้	13
14		M	25	K	เต่าอิน	3.	เต่าอิน	3	เต่าอิน	3.
15		M	36	K.	พะด่าน	3	พะด่าน	3	พะด่าน	3
16		M	47	K.	ผ่านกู้	4	ผ่านกู้	4	ผ่านกู้	4
17		M.	30	K.	พะด่าน	3	พะด่าน	3	พะด่าน	3
18		M	23	K.	หัวเวี๋ย	3	หัวเวี๋ย	3	หัวเวี๋ย	3
19		F	43.	I.	มอละแลม่ง	3	มอละหม่ง	3	มอละหม่ง	3
20		F	20	K + V	ไลบอง	2:3	ไลบอง [illegible]	2.3	ไลบอง	
